# Energy Metabolism, Metabolite, and Inflammatory Profiles in Human Ex Vivo Adipose Tissue Are Influenced by Obesity Status, Metabolic Dysfunction, and Treatment Regimes in Patients with Oesophageal Adenocarcinoma

**DOI:** 10.3390/cancers15061681

**Published:** 2023-03-09

**Authors:** Fiona O’Connell, Eimear Mylod, Noel E. Donlon, Aisling B. Heeran, Christine Butler, Anshul Bhardwaj, Sinead Ramjit, Michael Durand, Gerard Lambe, Paul Tansey, Ivan Welartne, Kevin P. Sheahan, Xiaofei Yin, Claire L. Donohoe, Narayanasamy Ravi, Margaret R. Dunne, Lorraine Brennan, John V. Reynolds, Helen M. Roche, Jacintha O’Sullivan

**Affiliations:** 1 Department of Surgery, Trinity St. James’s Cancer Institute and Trinity Translational Medicine Institute, St. James’s Hospital and Trinity College Dublin, D08 W9RT Dublin, Ireland; 2 Cancer Immunology and Immunotherapy Group, Department of Surgery, Trinity College Dublin, St. James’s Hospital, D08 W9RT Dublin, Ireland; 3 Department of Radiology, St. James’s Hospital, D08 NHY1 Dublin, Ireland; 4 Department of Radiology, Beaumont Hospital, D02 YN77 Dublin, Ireland; 5 UCD School of Agriculture and Food Science, Conway Institute of Biomolecular and Biomedical Research, University College Dublin, Belfield, D04 V1W8 Dublin, Ireland; 6 School of Chemical & Biopharmaceutical Sciences, Technological University Dublin, Tallaght, D07 EWV4 Dublin, Ireland; 7 Nutrigenomics Research Group, UCD Conway Institute, School of Public Health, Physiotherapy and Sports Science, University College Dublin, D04 C1P1 Dublin, Ireland; 8 Institute for Global Food Security, School of Biological Sciences, Queens University Belfast, Belfast BT9 5DL, UK

**Keywords:** oesophageal adenocarcinoma, obesity, metabolic dysfunction, metabolism, inflammation, treatment response, adipose tissue

## Abstract

**Simple Summary:**

Oesophageal adenocarcinoma (OAC) is a poor prognosis cancer with limited response rates to current treatment modalities and is strongly linked to obesity status and metabolic dysfunction. This study for the first time has conducted a detailed assessment of adipose tissue metabolism, as well as assessing adipose secreted pro-inflammatory, metabolite, and lipid profiles to determine whether these profiles correlate with significant clinical parameters in OAC patients including obesity, metabolic dysfunction, previous treatment exposure, and tumour regression grades. Overall, in this study, increases in metabolic profiles linked with oxidative phosphorylation, pro-inflammatory cytokines, and metabolites associated with aiding tumorigenesis have been identified in the most viscerally obese OAC patients and in patients with metabolic dysfunction. These results raise the question of whether targeting these altered signalling mechanisms could aid current treatment strategies.

**Abstract:**

Oesophageal adenocarcinoma (OAC) is a poor prognosis cancer with limited response rates to current treatment modalities and has a strong link to obesity. To better elucidate the role of visceral adiposity in this disease state, a full metabolic profile combined with analysis of secreted pro-inflammatory cytokines, metabolites, and lipid profiles were assessed in human ex vivo adipose tissue explants from obese and non-obese OAC patients. These data were then related to extensive clinical data including obesity status, metabolic dysfunction, previous treatment exposure, and tumour regression grades. Real-time energy metabolism profiles were assessed using the seahorse technology. Adipose explant conditioned media was screened using multiplex ELISA to assess secreted levels of 54 pro-inflammatory mediators. Targeted secreted metabolite and lipid profiles were analysed using Ultra-High-Performance Liquid Chromatography coupled with Mass Spectrometry. Adipose tissue explants and matched clinical data were collected from OAC patients (*n* = 32). Compared to visceral fat from non-obese patients (*n* = 16), visceral fat explants from obese OAC patients (*n* = 16) had significantly elevated oxidative phosphorylation metabolism profiles and an increase in Eotaxin-3, IL-17A, IL-17D, IL-3, MCP-1, and MDC and altered secretions of glutamine associated metabolites. Adipose explants from patients with metabolic dysfunction correlated with increased oxidative phosphorylation metabolism, and increases in IL-5, IL-7, SAA, VEGF-C, triacylglycerides, and metabolites compared with metabolically healthy patients. Adipose explants generated from patients who had previously received neo-adjuvant chemotherapy (*n* = 14) showed elevated secretions of pro-inflammatory mediators, IL-12p40, IL-1α, IL-22, and TNF-β and a decreased expression of triacylglycerides. Furthermore, decreased secreted levels of triacylglycerides were also observed in the adipose secretome of patients who received the chemotherapy-only regimen FLOT compared with patients who received no neo-adjuvant treatment or chemo-radiotherapy regimen CROSS. For those patients who showed the poorest response to currently available treatments, their adipose tissue was associated with higher glycolytic metabolism compared to patients who had good treatment responses. This study demonstrates that the adipose secretome in OAC patients is enriched with mediators that could prime the tumour microenvironment to aid tumour progression and attenuate responses to conventional cancer treatments, an effect which appears to be augmented by obesity and metabolic dysfunction and exposure to different treatment regimes.

## 1. Introduction

Oesophageal adenocarcinoma (OAC) is an aggressive disease associated with a poor prognosis and a five-year survival rate of approximately 20% [1], with current projections indicating that the incidence of this disease is increasing [2]. Currently, the standard of care for treatment involves neo-adjuvant treatment (treatment prior to surgery) with either chemotherapy alone including the FLOT regimen (consisting of 5FU, Folinic acid, Oxaliplatin, Docetaxel) or combination chemo-radiotherapy such as the CROSS regimen (consisting of Carboplatin, paclitaxel with concurrent 41.4 Gy radiation), for locally advanced tumours [3]. Unfortunately, only approximately 30% of patients show a complete response to these current treatment modalities, leaving a large proportion of patients with no therapeutic gain and a possible delay to surgery [4,5]. Furthermore, large-scale epidemiological studies demonstrate a consistent and compelling association between the risk of cancer development/progression and elevated body mass index (BMI) for many gastrointestinal cancers including OAC, with this cancer having one of the strongest associations with obesity [6,7,8]. This makes it an exemplary model for studying the influence of obesity on cancer and the role of the adipose tissue microenvironment in this setting.

Numerous factors are associated with the obese adipose tissue microenvironment such as chronic low-grade inflammation, angiogenesis, fibrosis, and the altered secretions of cells, all implicated in the progression and recurrence of cancer [9,10]. One of the significant effects of the obese adipocyte secretome is the release of a series of pro-inflammatory factors, leading to a local environment that is primed to aid tumour development and progression [11]. The altered milieu of the obese tumour environment has been extensively shown to have detrimental effects on the anti-tumour response, diminishing immune cell function and treatment efficacy [12,13,14]. It has been previously shown that adipose-conditioned media from oesophageal cancer patients increases radiosensitivity [15], and this could be linked to the differential expression of leptin receptors and its associated biology in driving inflammation in the adipose tissue microenvironment [16].

Currently, discrepancies are reported in the literature on whether obesity diminishes [17] or ameliorates [15] treatment resistance and whether obese individuals possess an enhanced survival benefit compared with their non-obese counterparts [18], which highlights the importance of identifying the underlying biological mechanisms which play a role in this setting in the complex adipose tissue microenvironment. Previous work has observed elevated oxidative phosphorylation in visceral adipose tissue compared with subcutaneous adipose tissue [16], and adipocytes derived from metabolically unhealthy obese individuals show elevated mitochondrial response profiles [19]. Whilst the role of tumour explant energy metabolism in OAC treatment response has been reported [20], the energy metabolism profiles of visceral adipose tissue between obese and non-obese OAC patients and the influence of its secretome on the cancer cell and immune cell function is still largely unknown.

For the first time, this study aims to better characterise the adipose tissue microenvironment using real-time energy metabolism profiles, profiling the secreted inflammatory environment, and assessing altered metabolites and lipid profiles of human ex vivo adipose explants. These profiles were examined based on obesity status, metabolic dysfunction, previous treatment exposure, and treatment responses in OAC patients. Overall, the profiling data and clinical correlations described in this study suggest the adipose microenvironment as well as potentiating a pro-tumorigenic milieu may also be linked with the efficacy of current standard-of-care cancer treatments. With the knowledge that the obesity epidemic is projected to increase, with 50% of the Western world population being obese by 2030 [21], this research endeavours to address an exigent question: what influence might the adipose microenvironment possess in the cancer–obesity link?

## 2. Materials and Methods

### 2.1. Ethics Statement and Patient Recruitment

Ethical approval was granted by the St James’s Hospital/AMNCH ethical review board (Ethics number: REC_2019-07 List 25(27)), and written informed consent was collected from all patients in this study. Thirty-two patients were recruited within the period between 1 December 2019 and 30 January 2022, and the patient demographics are listed in Table 1. All fresh adipose tissues were taken at the start of the surgical tumour resection procedure from OAC or OGJ (oesophagogastric junctional adenocarcinoma) patients being treated with curative intent.

### 2.2. Clinical Data Collation and Assessment

Obesity was defined using visceral fat area (VFA) measurements with a cut-off value for VFA of 163.8 cm^2^ for males and 80.1 cm^2^ for females as previously categorised [22]. Metabolic dysfunction was defined if a patient had 3 or more of the following criteria: visceral obesity (as assessed with the VFA cut-offs mentioned above), previously diagnosed type 2 diabetes or impaired fasting glucose, triglycerides ≥ 1.7 mmol/L or interventional treatment for high triglycerides; high-density lipoprotein cholesterol or interventional treatment for low HDL, systolic blood pressure ≥ 130 mmHg and/or diastolic blood pressure ≥ 85 mmHg or treatment for hypertension [22,23]. Previous treatment was classified as a patient receiving either neo-adjuvant chemotherapy only (FLOT regimen) or chemo-radiotherapy (CROSS regimen), patients who received no neo-adjuvant treatment prior to surgery were classified as treatment naïve. Histological assessment of resected tumours was conducted by a pathologist, using the Mandard tumour regression grade to assess patient’s response to neo-adjuvant treatment; therefore, no TRG scoring is available for treatment naïve patients who did not receive chemotherapy or chemo-radiotherapy. The clinical data summary is listed in Table 1.

### 2.3. Seahorse Analysis of Metabolic Profiles from Adipose Tissue Explants and Generation of Adipose Conditioned Media (ACM)

Fresh omental tissue was collected from theatre and processed within 30 min by dissecting it into pieces weighing approximately 20 mg. Tissue was plated in triplicates in 1 mL of M199 (Gibco, Thermofisher, Waltham, MA, USA) supplemented with 0.1% gentamicin (Lonza, Switzerland), in a 24-well plate (Sarstedt, Nümbrecht, Germany). Adipose explants were cultured for 24 h at 37 °C and 5% carbon dioxide in a humidified incubator (Thermofisher, MA, USA). In the last hour of culture, adipose tissue and ACM were transferred to an islet capture microplate with capture screens (Agilent Technologies, Santa Clara, CA, USA) and incubated in a non-CO_2_ incubator at 37 °C (Whitley, West Yorkshire, UK) prior to analysis. Seahorse Xfe24 analyser was used to assess metabolic profiles in adipose explants (Agilent Technologies, CA, USA). Following a 12 min equilibrate step, three basal measurements of OCR (Oxygen Consumption Rate) and ECAR (Extracellular Acidification Rate) were taken over 24 min consisting of three repeats of the following sequence “mix (3 min)/wait (2 min)/measurement (3 min)” to establish basal respiration. Adipose Conditioned Media (ACM) was extracted in a sterile environment and tissue was weighed using a benchtop analytical balance (Radwag, Radom, Poland) and snap frozen. All samples were then stored at −80 °C for further processing.

### 2.4. Multiplex ELISA

The collected Adipose Conditioned Media (ACM) was processed according to MSD (Meso Scale Discovery, Rockville, Maryland, USA) multiplex protocol. To assess angiogenic, vascular injury, pro-inflammatory, and cytokine and chemokine secretions from ACM, a 54-plex ELISA kit separated across 7 plates was used (Meso Scale Discovery, Rockville, Maryland, USA). The multiplex kit was used to quantify the secretions of CRP, Eotaxin, Eotaxin-3, FGF(basic), Flt-1, GM-CSF, ICAM-1, IFN-γ, IL-10, IL-12/IL-23p40, IL-12p70, IL-13, IL-15, IL-16, IL-17A, IL-17A/F, IL-17B, IL-17C, IL-17D, IL-1RA, IL-1α, IL-1β, IL-2, IL-21, IL-22, IL-23, IL-27, IL-3, IL-31, IL-4, IL-5, IL-6, IL-7, IL-8, IL-8 (HA), IL-9, IP-10, MCP-1, MCP-4, MDC, MIP-1α, MIP-1β, MIP-3α, PlGF, SAA, TARC, Tie-2, TNF-α, TNF-β, TSLP, VCAM-1, VEGF-A, VEGF-C, and VEGF-D from ACM. All assays were run as per the manufacturer’s recommendation, and an overnight supernatant incubation protocol was used for all assays except Angiogenesis Panel 1 and Vascular Injury Panel 2, which were run according to the same-day protocol. ACM was run undiluted for all assays except Vascular Injury Panel 2, where a one-in-four dilution was used, as per previous optimization experiments. Assays were run on a MESO QuickPlex SQ 120, and all analyte concentrations were calculated using Discovery Workbench software (version 4.0). Secretion data for all factors were normalized to adipose post-incubation weight and expressed as pg/mL per gram of adipose tissue.

### 2.5. Metabolomic and Lipidomic Screening

ACM was analysed using a targeted metabolomic platform and was prepared according to the MxP^®^ Quant 500 assay manual (Biocrates Life Sciences, Innsbruck, Austria). Samples were dried and derivatised using derivatization solution (5% phenyl isothiocyanate in ethanol/water/pyridine (volume ratio 1/1/1)) and incubated for 1 h at room temperature and then dried under nitrogen for 1 h. After the addition of 300 μL of 5 mM ammonium acetate in methanol, the plate was shaken for 30 min and then centrifuged at 500 g for 2 min, and then 150 μL of high-performance liquid chromatography (HPLC)-grade water was added for liquid chromatography-tandem mass spectrometry (LC-MS/MS) analysis. Additionally, 10 μL of the eluate was diluted with 490 μL of methanol running solvent for flow injection analysis tandem mass spectrometry (FIA-MS/MS) analysis. Samples were analysed using the Sciex ExionLC series UHPLC system coupled to a Sciex QTRAP 6500+ mass spectrometer. The UHPLC columns (Biocrates Life Sciences, Innsbruck, Austria) were installed, and mobile phases A and B were 100% water and 95% acetonitrile (both added 0.2% formic acid), respectively. In the LC-MS/MS analysis, amino acids (*n* = 20) and amino acid-related (*n* = 30), bile acids (*n* = 14), biogenic amines (*n* = 9), carboxylic acids (7), hormones and related (*n* = 4), indoles and derivatives (*n* = 4), nucleobases and related (*n* = 2), fatty acids (*n* = 12), trigonelline, trimethylamine N-oxide, p-Cresol sulphate, and choline were quantified. Lipid classes such as lysophosphatidylcholines (*n* = 14), phosphatidylcholines (*n* = 76), sphingomyelins (*n* = 15), ceramides (*n* = 28), dihydroceramides (*n* = 8), hexosylceramides (*n* = 19), dihexosylceramides (*n* = 9), trihexosylceramides (*n* = 6), cholesteryl esters (*n* = 22), diglycerides (*n* = 44), and triglycerides (*n* = 242) were quantified using the FIA-MS/MS analysis. Furthermore, acylcarnitines (*n* = 40) and the sum of hexose were also quantified using the FIA-MS/MS analysis. The multiple reaction monitoring (MRM) method, which was optimized by Biocrates Life Sciences, was applied to identify and quantify all metabolites.

### 2.6. Statistical Analysis

All statistics were conducted using GraphPad Prism 9.0 (GraphPad Software, San Diego, CA, USA). A significance level of *p* < 0.05 was used in all analyses and all *p*-values reported were two-tailed. The Mann–Whitney test was used for the continual non-parametric dependent variable analysis of data with two groups. For more than two groups, the Kruskal–Wallis test with Dunn’s correction was used. Details of specific statistical tests are given in each corresponding figure legend. To determine if protein expression levels identified using 54-plex ELISA correlated with patient clinical factors, Spearman correlations were carried out using R software version 3.6.2 [24]. Correlations were generated using the R package ‘Hmisc’ version 4.4-0 [25]. Graphical representations of correlations were generated with the R package ‘corrplot’ version 0.84 [26]. All correlations with an associated *p*-value < 0.05 were considered statistically significant. The Holm–Bonferroni post hoc correction was used to control for multiple comparison testing during correlation analysis.

## 3. Results

### 3.1. Increased Oxidative Phosphorylation Metabolism and Elevated Secreted Pro-Inflammatory Mediators Were Observed in Adipose Tissue Explants from Viscerally Obese Patients

To assess whether obesity determined using visceral fat area alters metabolic and secreted profiles of visceral omental ex vivo explants from obese OAC patients (*n* = 16) and non-obese patients (*n* = 16), the Agilent Seahorse Xfe24 analyser was used to assess real-time metabolic parameters. To further assess the influence of these clinical parameters on this explant model, the matched ACM secretome was evaluated using MSD 54 plex ELISA and metabolomic and lipidomic profiling.

**Figure 1:**
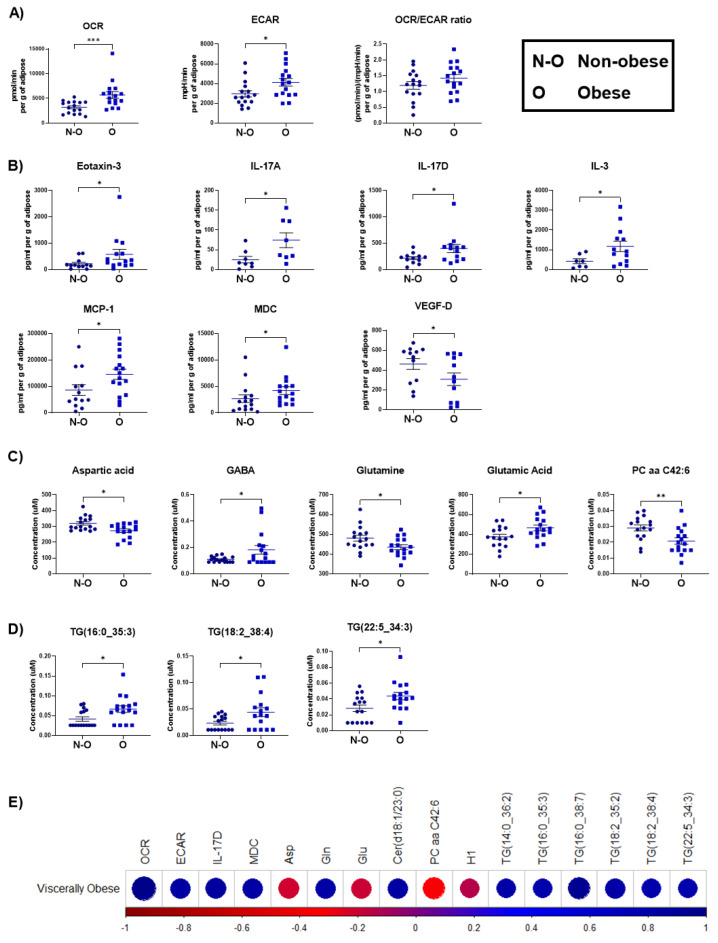
Altered OCR, ECAR, pro-inflammatory cytokines, and metabolites in adipose explants from obese OAC patients compared with non-obese patients. (**A**) OCR, ECAR, and OCR:ECAR ratio (Mann–Whitney test). (**B**) Eotaxin-3, IL-17A, IL-17D, IL-3, MCP-1, MDC, and VEGF-D (Mann–Whitney test). (**C**) Aspartic acid, GABA, Glutamine, Glutaric acid, and PC aa C42:6 (Mann–Whitney test). (**D**) TG(16:0_35:3), TG(18:2_38:4), and TG(22:5_34:3) (Mann–Whitney test). (**E**) Correlation plot showing only significant correlations (*p* < 0.05) between experimental data and visceral obesity (Spearman correlation, blue indicates positive correlations and red indicates inverse negative correlations). The Holm–Bonferroni post hoc correction was used to control for multiple comparison testing. All data expressed as mean ± SEM, obese *n* = 16, non-obese *n* = 16, * *p* < 0.05, ** *p* < 0.01, *** *p* < 0.001.

Significant increases were observed in OCR (*p* = 0.0009) and ECAR (*p* = 0.0260) (Figure 1A) profiles in visceral adipose explants derived from obese oesophageal cancer patients compared with non-obese patients. Increased secretions of cytokines Eotaxin-3, IL-17A, IL-17D, IL-3, MCP-1, and MDC and a decrease in the secretion of VEGF-D (*p* = 0.0356, 0.0281, 0.0457, 0.0369, 0.0358, and 0.0408, respectively) (Figure 1B) were observed in adipose explants from obese patients compared with their non-obese counterparts. A decreased expression of metabolites Aspartic acid, Glutamine, and PC aa C42:6, (*p* = 0.0240, 0.0280, and 0.0063, respectively) (Figure 1C) was observed in the secretome of adipose explants from obese patients compared with their non-obese counterparts. An elevated expression of metabolites GABA, Glutamic acid, TG (16:0_35:3), TG(18:2_38:4), TG(22:5_34:3) (0.0419, 0.0343, 0.0315, 0.0468, 0.0316 respectively) (Figure 1C,D) was additionally observed in the adipose secretome from obese patients compared with non-obese patients. Significant correlations observed between the experimental data and visceral fat area were visualised using corrplot (Figure 1E), and the associated R numbers and *p*-values are detailed in Table 2.

### 3.2. Adipose Explants Derived from OAC Patients with Metabolic Dysfunction Show Increased Oxidative Phosphorylation Associated Metabolism and Secreted Pro-Inflammatory Mediators

Aberrant biological mechanisms such as obesity, diabetes, high triacylglycerides, high cholesterol, and high blood pressure have all been identified as contributors to the development of metabolic syndrome, a pro-inflammatory condition to aid cancer progression [27]. In this study, to assess the influence of metabolic dysfunction, on visceral adipose explants from metabolically healthy (*n* = 17) and metabolically dysfunctional (*n* = 15) OAC patients, four profiling assays looking at metabolism, secreted pro-inflammatory mediators, and lipid/metabolite profiles were used.

**Figure 2:**
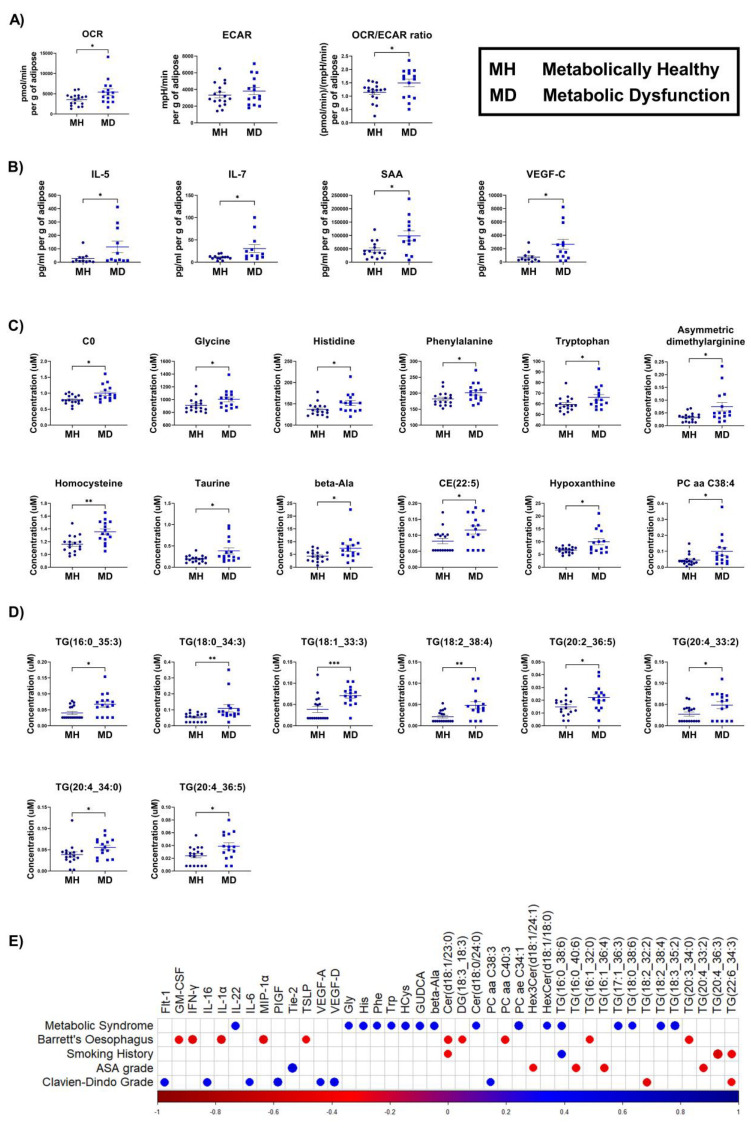
Altered OCR, OCR:ECAR ratio, pro-inflammatory cytokines, and metabolites in adipose explants from OAC patients with metabolic dysfunction compared with metabolically healthy OAC patients. (**A**) OCR, ECAR, and OCR:ECAR ratio (Mann–Whitney test). (**B**) IL-5, IL-7, SAA, and VEGF-C (Mann–Whitney test). (**C**) C0, Glycine, Histidine, Phenylalanine, Tryptophan, Asymmetric dimethylarginine, Homocysteine, Hypoxanthine, Taurine, beta-Ala, CE(22:5), and PC aa C38:4 (Mann–Whitney test). (**D**) TG(16:0_35:3), TG(18:0_34:3), TG(18:1_33:3), TG(18:2_38:4), TG(20:2_36:5), TG(20:4_33:2), TG(20:4_34:0), and TG(20:4_36:5) (Mann–Whitney test). (**E**) Correlation plot showing only significant correlations (*p* < 0.05) between experimental data and metabolic syndrome, Barrett’s oesophagus, smoking history, ASA grade, and Clavian–Dindo grade (Spearman correlation, blue indicates positive correlations and red indicates inverse negative correlations). The Holm–Bonferroni post hoc correction was used to control for multiple comparison testing. All data expressed as mean ± SEM, metabolic syndrome *n* = 15, metabolically healthy *n* = 17, * *p* < 0.05, ** *p* < 0.01, *** *p* < 0.001.

In adipose explants derived from patients with clinically annotated metabolic dysfunction, significant increases in OCR (*p* = 0.0486) and the OCR/ECAR ratio (*p* = 0.0402) were identified (Figure 2A) in metabolic profiles compared with metabolically healthy patients. Furthermore, significant increases in secreted cytokines including IL-5, IL-7, SAA, and VEGF-C (*p* = 0.0473, 0.0257, 0.0253, 0.0301) (Figure 2B) were detected in metabolically unhealthy patients compared with metabolically healthy patients. The following metabolites were also identified to be significantly elevated in the adipose secretome of patients with metabolic dysfunction: C0, Glycine, Histidine, Phenylalanine, Tryptophan, Asymmetric dimethylarginine, Homocysteine, Hypoxanthine, Taurine, beta-Ala, CE(22:5), and PC aa C38:4 (*p* = 0.0137, 0.0299, 0.0275, 0.0452, 0.0372, 0.0122, 0.0016, 0.0299, 0.0172, 0.0219, 0.0327, and 0.0291, respectively) (Figure 2C). Following the lipidomic analysis, triglycerides including TG(16:0_35:3), TG(18:0_34:3), TG(18:1_33:3), TG(18:2_38:4), TG(20:2_36:5), TG(20:4_33:2), TG(20:4_34:0), and TG(20:4_36:5) (*p* = 0.0105, 0.0036, 0.0009, 0.0025, 0.0152, 0.0327, 0.0321, and 0.0265, respectively) (Figure 2D) were also seen to be elevated in the adipose secretome of OAC patients with metabolic dysfunction compared with patients who were metabolically healthy. Significant correlations were also observed between the experimental data with metabolic dysfunction, Barrett’s oesophagus, smoking history, the ASA grade, and the Clavien–Dindo grade, which were visualised using corrplot (Figure 2E), and the associated R numbers and *p*-values are detailed in supplemental Table S1.

### 3.3. Adipose Explants from Patients Receiving the FLOT Chemotherapy Regimen Showed Increased Oxidative Phosphorylation and Pro-Inflammatory Mediators and Decreased Triacylglycerides

Patients were classified as treatment naïve if they had received no treatment prior to surgical intervention (*n* = 10), FLOT if they had received neo-adjuvant chemotherapy only regimen FLOT (*n* = 14), or CROSS if they received neo-adjuvant chemo-radiotherapy regimen CROSS (=8). The four experimental assays looking at metabolism, secreted pro-inflammatory mediators, and lipid/metabolite profiles were utilised to identify whether any associations were observed between previous treatment exposure and adipose tissue functionality.

**Figure 3:**
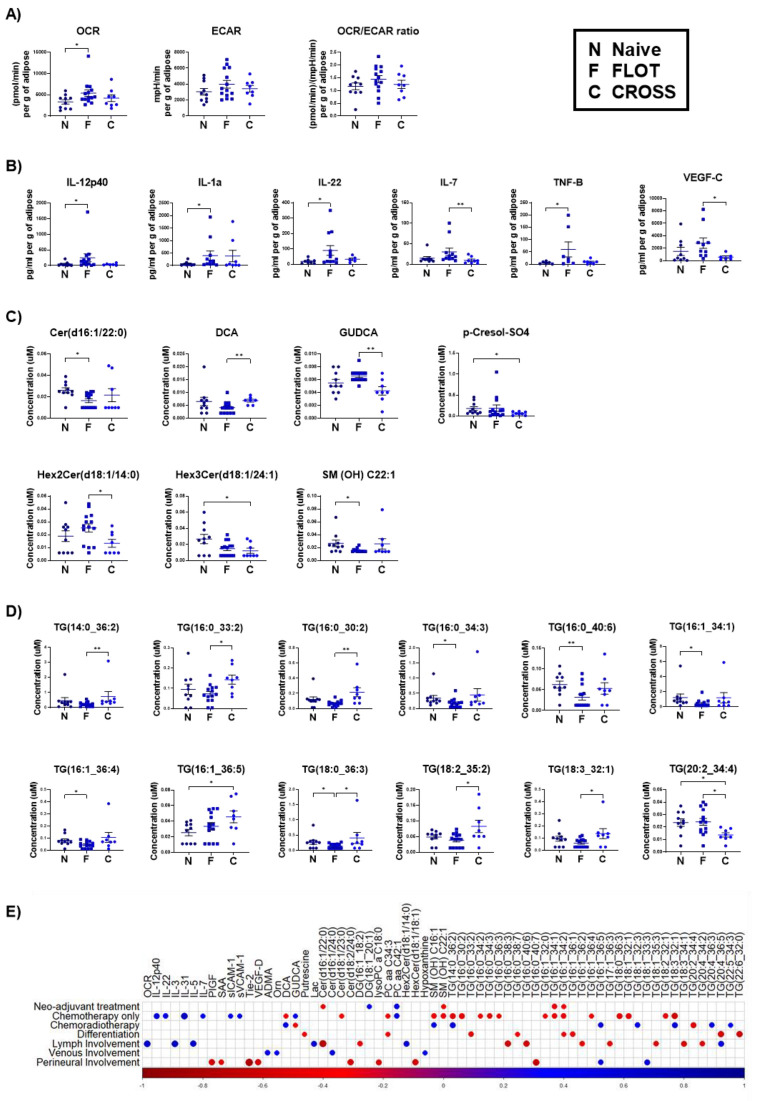
Alterations in OCR, pro-inflammatory cytokines and metabolites in adipose explants from OAC patients correlates with previous treatment exposure (**A**) OCR, ECAR, and OCR:ECAR ratio (Kruskal–Wallis with Dunn’s correction). (**B**) IL-12p40, IL-1α, IL-22, IL-7, TNF-β, and VEGF-C (Kruskal–Wallis with Dunn’s correction). (**C**) Cer(d16:1/22:0), DCA, GUDCA, p-Cresol-SO4, Hex2Cer(d18:1/14:0), Hex3Cer(d18:1/24:1), SM (OH) C22:1, TG(14:0_36:2), and TG(16:0_33:2), TG(16:0_30:2) (Kruskal–Wallis with Dunn’s correction). (**D**) TG(16:0_34:3), TG(16:0_40:6), TG(16:1_34:1), TG(16:1_36:4), TG(16:1_36:5), TG(18:0_36:3), TG(18:2_35:2), TG(18:3_32:1), and TG(20:2_34:4) (Kruskal–Wallis with Dunn’s correction). (E) Correlation plot showing only significant correlations (*p* < 0.05) between experimental data and with neo-adjuvant treatment, chemotherapy only, chemo-radiotherapy differentiation, lymph involvement, venous involvement, and perineural involvement (Spearman correlation, blue indicates positive correlations and red indicates inverse negative correlations). The Holm–Bonferroni post hoc correction was used to control for multiple comparison testing. All data expressed as mean ± SEM, Naïve *n* = 10, FLOT *n* = 14, CROSS = 8, * *p* < 0.05, ** *p* < 0.01.

Increased OCR (*p* = 0.0338) (Figure 3A) metabolic profiles were observed in adipose explants derived from patients who had previously been treated with the FLOT chemotherapy regimen compared with patients who did not have neo-adjuvant treatment. Increased secretion of cytokines IL-12p40, IL-1α, IL-22, and TNF-β (*p* = 0.0340, 0.0426, 0.0350, and 0.0392, respectively) (Figure 3B) were observed in the adipose secretome of patients previously treated with FLOT compared with patients who were treatment-naïve. Decreased secretion of cytokines IL-7 and VEGF-C (*p* = 0.0061 and 0.0231, respectively) (Figure 3B) were observed in the adipose explants from patients who had received neo-adjuvant chemo-radiotherapy regimen CROSS compared with FLOT. Metabolites including Cer(d16:1/22:0), SM (OH) C22:1, TG(16:0_34:3), TG(16:0_40:6), TG(16:1_34:1), TG(16:1_36:4), and TG(18:0_36:3) (*p* = 0.0106, 0.0165, 0.0375, 0.0092, 0.0138, 0.0470, and 0.0370, respectively) (Figure 3C,D) were significantly decreased in the secretome of the adipose explants derived from patients who had previously been treated with the FLOT regimen compared with treatment-naïve patients. Significant decreases were observed in metabolites p-Cresol-SO4, Hex3Cer(d18:1/24:1), and TG(20:2_34:4) (*p* = 0.0333, 0.0422, and 0.0177, respectively) (Figure 3C,D) whilst metabolite TG(16:1_36:5) (*p* = 0.0364) was significantly increased in the adipose secretome of patients who had previously received the CROSS regimen compared with patients who were treatment-naïve. Metabolites GUDCA, Hex2Cer(d18:1/14:0), and TG(20:2_34:4) (*p* = 0.0031, 0.0173, and 0.0106, respectively) (Figure 3C,D) were all observed to be decreased and metabolites DCA, TG(14:0_36:2), TG(16:0_33:2), TG(16:0_30:2), TG(18:0_36:3), and TG(18:2_35:2), TG(18:3_32:1) (*p* = 0.0069, 0.0040, 0.0177, 0.0010, 0.0406, 0.0348, and 0.0131, respectively) (Figure 3C,D) were observed as increased in the adipose secretome of patients who received the chemo-radiotherapy CROSS regimen compared with patients who received the chemotherapy only FLOT regimen. In particular, the metabolite TG(18:0_36:3) was decreased in the adipose secretome of patients receiving the FLOT regimen compared with both the treatment-naïve patients and patients receiving the CROSS regimen (*p* = 0.0370 and 0.0406, respectively) (Figure 3D) whilst metabolite TG(20:2_34:4) was decreased in the adipose secretome of patients receiving the CROSS regimen compared with both treatment-naïve patients and patients receiving the FLOT regimen (*p* = 0.0177, 0.0106) (Figure 3D). Significant correlations observed between the experimental data with neo-adjuvant treatment, chemotherapy only, chemoradiotherapy, tumour differentiation, lymph involvement, venous involvement, and perineural involvement were visualised using corrplot (Figure 3E), and the associated R numbers and *p*-values are detailed in Supplemental Tables S2 and S3.

### 3.4. Increased ECAR and Altered Metabolites Are Observed in Adipose Explants from OAC Patients with Increasing Tumour Regression Grades

Tumour regression grading was used to assess patients’ response to treatment received; therefore, experimental data within this section only relates to patients who received neo-adjuvant treatment prior to surgery, and patients who did not receive treatment prior to surgery were excluded from this analysis. TRG 1–2 (*n* = 6) indicate patients who had a complete or good response to therapy, patients with TRG 3 (*n* = 8) showed an intermediate response, and patients with TRG 4–5 (*n* = 6) had a poor response. The association of this staging with adipose explant metabolism and secretome was assessed using the four profiling assays looking at metabolism, secreted pro-inflammatory mediators, and lipid/metabolite profiles.

**Figure 4:**
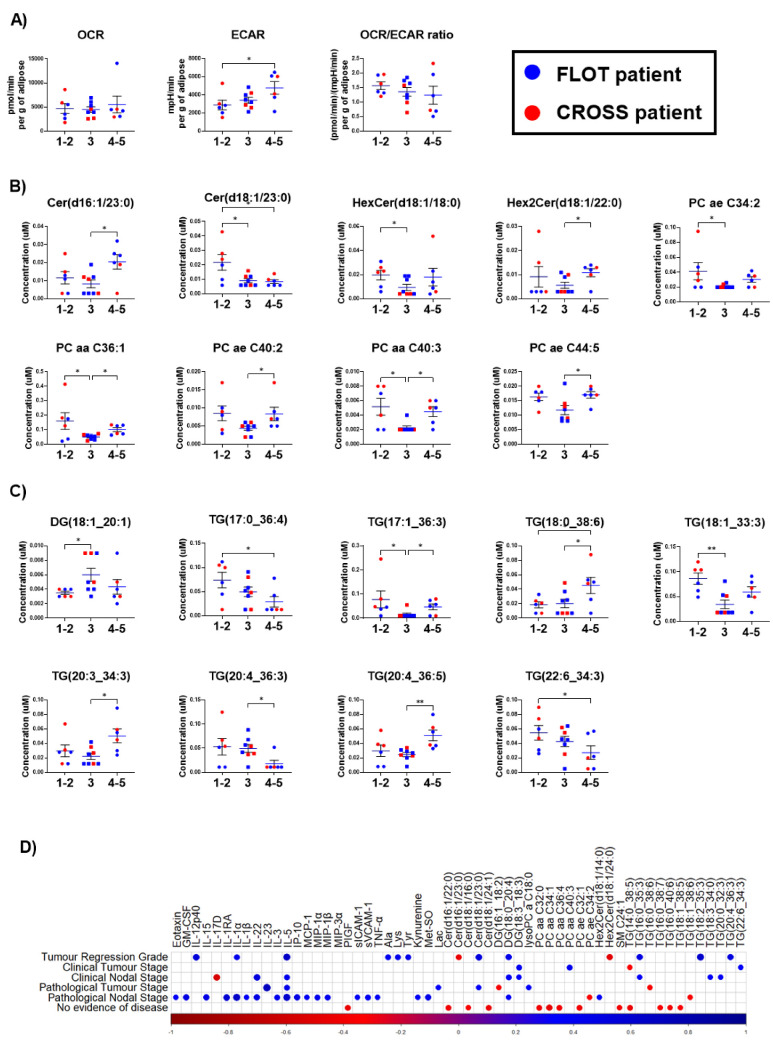
Increased ECAR and altered metabolites are observed in adipose explants from OAC patients with increasing tumour regression grades. (**A**) OCR, ECAR, and OCR:ECAR ratio (Kruskal–Wallis with Dunn’s correction). (**B**) Cer(d16:1/23:0), Cer(d18:1/23:0), PC aa C36:1, PC aa C40:3, PC ae C34:2, PC ae C40:2, PC ae C44:5, Hex2Cer(d18:1/22:0), and HexCer(d18:1/18:0) (Kruskal–Wallis with Dunn’s correction). (**C**) DG(18:1_20:1), TG(17:0_36:4), TG(17:1_36:3), TG(18:0_38:6), TG(18:1_33:3), TG(20:3_34:3), TG(20:4_36:3), TG(20:4_36:5), and TG(22:6_34:3) (Kruskal–Wallis with Dunn’s correction). (**D**) Correlation plot showing only significant correlations (*p* < 0.05) between experimental data and tumour regression grade, clinical tumour stage, clinical nodal stage, pathological tumour stage, pathological nodal stage, and no evidence of disease (Spearman correlation, blue indicates positive correlations and red indicates inverse negative correlations). The Holm–Bonferroni post hoc correction was used to control for multiple comparison testing. All data expressed as mean ± SEM, TRG 1–2 *n* = 6, TRG 3 *n* = 8, TRG 4–5 *n* = 6, * *p* < 0.05, ** *p* < 0.01. Blue symbols identify patients who received neo-adjuvant chemotherapy (FLOT) and red symbols identify patients who received neo-adjuvant chemo-radiotherapy (CROSS).

Significantly increased ECAR profiles (*p* = 0.0248) (Figure 4A) were observed in the secretome of adipose explants derived from patients with TRG scoring of 4–5 compared with patients with TRG scorings of 1–2. A decreased expression of metabolites Cer(d18:1/23:0), PC aa C36:1, PC aa C40:3, PC ae C34:2, HexCer(d18:1/18:0), TG(17:1_36:3), and TG(18:1_33:3) (*p* = 0.0452, 0.0445, 0.0183, 0.0318, 0.0269, 0.0262, and 0.0081, respectively) (Figure 4B,C) and an increased expression of DG(18:1_20:1) (*p* = 0.0393) (Figure 4C) were observed in the adipose secretome from patients with a TRG of 3 compared with patients with TRG scorings of 1–2. A decreased expression of metabolites Cer(d18:1/23:0), TG(17:0_36:4), and TG(22:6_34:3) (*p* = 0.0409, 0.0249, and 0.0451, respectively) (Figure 4B,C) and an increased expression of TG(18:0_38:6) (*p* = 0.0434) (Figure 4C) were observed in the secretome of adipose explants derived from patients with a TRG of 4–5 compared with patients with TRG scorings of 1–2. A significant decrease was observed in metabolites Cer(d16:1/23:0), PC aa C36:1, PC aa C40:3, PC ae C40:2, PC ae C44:5, Hex2Cer(d18:1/22:0), TG(17:1_36:3), TG(18:0_38:6), TG(20:3_34:3), and TG(20:4_36:5) (*p* = 0.0163, 0.0303, 0.0246, 0.0446, 0.0324, 0.0493, 0.0425, 0.0478, 0.0173, and 0.0046, respectively) (Figure 4B,C) and a significant increase in triglyceride TG(20:4_36:3) (*p* = 0.0252) (Figure 4C) were observed in the adipose secretome of patients with a TRG of 3 compared with patients with a TRG of 4–5. Of note, metabolite Cer(d18:1/23:0) (*p* = 0.0452, 0.0409) (Figure 4B) was decreased in the adipose secretome of patients with a TRG score of 3 and TRG of 4–5 compared with patients who had a TRG of 1–2. A decreased expression was also observed in TG(18:0_38:6) (*p* = 0.0434, 0.0478) (Figure 4C) in the secretome of adipose explants derived from patients with a TRG of 1–2 and a TRG of 3 compared with patients who possessed a TRG score of 4–5. It was also identified that metabolites including PC aa C36:1 (*p* = 0.0445, 0.0303), PC aa C40:3, (*p* = 0.0183, 0.0246), and TG(17:1_36:3) (*p* = 0.0262, 0.0425) (Figure 4B,C) had increased expression in the adipose secretome of patients with TRGs of 1–2 and 4–5, respectively, compared with patients with a TRG scoring of 3. Significant correlations observed between experimental data with tumour regression grade, clinical tumour stage, clinical nodal stage, pathological tumour stage, pathological nodal stage, and no evidence of disease were visualised using corrplot (Figure 4D), and the associated R numbers and *p*-values are detailed in Supplemental Tables S4 and S5.

## 4. Discussion

This study for the first time has conducted a detailed assessment of adipose tissue metabolism, as well as adipose secreted pro-inflammatory, metabolite, and lipid profiles and determined whether these profiles correlate with significant clinical parameters in OAC patients including obesity, metabolic dysfunction, previous treatment exposure and tumour regression grades. The recent literature has identified an elevated reliance of certain subtypes of cancers on oxidative phosphorylation rather than glycolysis [28]. Previous research from our group has shown an increased reliance on oxidative phosphorylation correlating with enhanced radioresistance in oesophageal cancer cells [29]. In this study, elevated OCR and ECAR metabolic profiles have been observed in adipose explants from obese OAC patients compared with their non-obese counterparts, indicating higher metabolic rates with increasing visceral adiposity. Previous work conducted in mouse models has shown that diminished oxidative phosphorylation function ameliorated obesity in mice on high-fat diets disrupting weight gain and improving glucose tolerance [30]. This study further shows that elevated OCR and OCR/ECAR ratio profiles were observed in patients classified with metabolic dysfunction compared with metabolically healthy patients. Previously, increased mitochondrial respiration was observed in adipocytes of metabolically unhealthy patients compared with metabolically healthy patients [19]. This shift towards utilisation of oxidative phosphorylation pathways within adipose tissue of obese and metabolically unhealthy individuals may provide insight into aberrant mitochondria function promoting pro-tumorigenic signalling. An elevated utilisation of oxidative phosphorylation pathways was observed in adipose tissue from our patients who received FLOT chemotherapy treatment compared with treatment-naïve patients. Previous research has shown that treatment with 5-FU and oxaliplatin upregulated genes associated with oxidative phosphorylation in mouse models [31]. This may be further augmented by elevated oxidative phosphorylation in adipose tissue sequestering chemotherapy within adipose tissue [32], which may hinder chemotherapy efficacy. Additionally, an increased reliance on glycolysis was observed in adipose explants with increasing TRGs (poor response). Studies have shown cancer cells co-cultured with adipose stromal cells upregulate glycolysis and mediated chemoresistance, which may support why elevated ECAR is observed only in adipose explants of patients with the poorest response to chemotherapy [33].

The secretome of adipose tissue comprises many pro-tumorigenic cytokines that are primed to aid cancer cell growth and survival [34]. Pro-inflammatory cytokines and metabolism are interlinked and essential in regulating adipocyte function and lipid metabolism, particularly in metabolic diseases [35]. Eotaxin-3, an adipose-associated cytokine, was elevated with obesity status in this study. Eotaxin-3 correlates with increased BMI [36] and is highly expressed in the circulation of OAC patients with longer survival rates [37]. However, Eotaxin-3 had also been reported to be highly expressed in tumour tissue in aggressive disease [38] and with increased macrophage infiltration [38]. Coupling this knowledge with the observed increased secretion of MDC and MCP-1 in this study, both macrophage-associated cytokines [39,40], the adipose secretome of obese individuals may aid the recruitment of macrophages and other immune cells to the adipose tissue leading to a diminished immune response in the local tumour microenvironment. The secretion of IL-17 A and IL-17 D was also increased in the adipose secretome of obese patients. Studies have linked elevated IL-17 in adipose tissue to increases in infiltrating immune cells [41,42] and poor prognosis [43], and the disruption of this pathway diminishes metastasis and enhances treatment response [44,45,46]. This cytokine may be a potential target in the obese tumour microenvironment. This study has also shown a decreased secretion of VEGF-D in the adipose secretome of obese OAC patients. Previous work has observed that while VEGF-A, VEGF-B, and VEGF-C showed increased circulating levels in obese individuals, the levels of VEGF-D were significantly diminished [47]. VEGF-D in particular has been linked with metastatic disease, and this diminished secretion may be amelioratory [48].

Increased expression of IL-5 in the adipose secretome of patients with metabolic dysfunction was identified. Previous studies in mouse models with suppressed IL-5/CD300f expression have shown decreased diet-induced weight gain and insulin resistance [49]. IL-5 facilitates lung metastasis [50] and targeting the IL-5 axis could ameliorate metabolic dysfunction symptoms. Previous findings have identified elevated circulating SAA in patients with metabolic syndrome, as was observed in the adipose secretome of metabolically unhealthy patients in this cohort [51]. SAA relates to the inflammatory processes, which play pivotal roles in both obesity and metabolic syndrome [52]. Elevated levels of IL-7 were observed in this study in the adipose secretome of metabolically unhealthy patients and in patients who received FLOT chemotherapy compared with CROSS chemo-radiotherapy. Previous work has shown that IL-7 overexpression in mouse models is associated with glucose and insulin resistance [53]. However, elevated IL-7 could prove beneficial in the context of FLOT chemotherapy as IL-7 can aid in T cell growth [54]. Recent studies have indicated that IL-7 could potentially re-sensitize tumours to cisplatin [55]. As previously mentioned, obese individuals have elevated circulating levels of VEGF-C, and the overexpression of VEGF-C in mouse models has been linked to increased weight gain and insulin resistance [47,56]. Consequently, this elevated secretion in the adipose secretome could potentiate metabolic disorder and prime the local tumour microenvironment for cancer cell survival. Elevated VEGF-C has also been linked to chemoresistance [57], and the increased secretion of VEGF-C observed within the adipose secretome of FLOT chemotherapy-treated patients compared to CROSS-treated patients may indicate a more systemic effect as this VEGF-C enriched adipose secretome could potentiate VEGF-C mediated metastasis [58].

Increased secretion of IL-22 and TNF-β was observed in the adipose secretome of patients who received FLOT chemotherapy compared with patients who received no neo-adjuvant therapy. IL-22, whilst possessing both pro- and anti-inflammatory effects, can promote cancer cell growth and enhance chemoresistance [59], allowing infiltration of M2-like macrophages in adipose tissue, and driving a systemic anti-tumour effect [60]. TNF-β promotes proliferation and invasion in cancer cell models [61]; however, the influence of chemotherapies on the functional role of TNF-β in adipose tissue is unknown. This study reports elevated levels of pro-inflammatory cytokines IL-12p40 and IL-1α in the adipose secretome of patients who received FLOT chemotherapy compared with treatment-naïve patients. Expression of IL-1 α in tumour tissue of gastric cancers has been shown to correlate with a more aggressive disease state and metastasis [62]; however, these cytokines can also act in immune cell recruitment [63].

Recent research has supported the utility of the metabolome in many diseases [64]. This study observed altered secretions in key metabolites involved in the glutamate/GABA-glutamine cycle. Increased secretion of GABA and glutamic acid (ionic form glutamate) and decreased secretion of glutamine were observed in the adipose secretome of obese patients compared with their non-obese counterparts. Previous research has also observed decreased glutamine and increased glutamate in the circulation of obese individuals [65,66]. Glutamine is effective in polarisation of anti-inflammatory M2-like macrophages, which may act as a potential therapeutic target to resolve the low-grade inflammation associated with obesity [66]. Aspartic acid was also decreased in the adipose secretome of obese patients compared with non-obese patients, which emulates previous research where decreased N-acetylaspartate was observed in patients with higher BMIs [67]. A combination of aspartic acid and glutamic acid may be capable of inducing tumour cell death [68]. Interestingly, these amino acids were decreased in the obese setting in our study. Elevated secreted levels of triacylglycerides TG(16:0_35:3), TG(18:2_38:4), and TG(22:5_34:3) were also detected in adipose tissue from obese OAC patients compared with non-obese. Accumulation of triacylglycerides has previously been reported in obese adipose tissue [69] and in cancers [70,71]. Elevated levels of asymmetric dimethylarginine, homocysteine, and hypoxanthine have been reported in individuals with metabolic syndrome [72,73,74]. This study also identified increased secretion of these amino acids in the adipose tissue of OAC patients that have metabolic dysfunction compared with metabolically healthy patients. Previous research has identified increased circulating levels of both homocysteine and hypoxanthine in cancer patients; however, the role they play in tumorigeneses or cancer cell survival is not fully understood [75,76]. However, reports of elevated asymmetric dimethylarginine in cancer have suggested it may attenuate apoptosis in response to stress and chemotherapy [77]. This study has also identified elevated levels of glycine and taurine in the adipose secretome of metabolically unhealthy versus metabolically healthy OAC patients. Elevated circulating levels of these amino acids should ameliorate the pro-inflammatory effects of metabolic syndrome and attenuate cancer cell proliferation as well as enhance the efficacy of chemotherapy [78,79].

Furthermore, p-Cresyl sulfate was decreased in the adipose secretome of patients who received the CROSS regimen compared with patients who received no neo-adjuvant therapy. p-Cresyl sulphate aids in cancer cell migration and epithelial–mesenchymal transition [80,81] and its diminished secretion in the adipose secretome of CROSS-treated patients could possess beneficial effects. In this study, DCA was observed to be increased in the adipose secretome of CROSS regimen-treated patients compared with FLOT-treated patients, whilst GUDCA (glycoursodeoxycholic acid) was observed to be decreased. DCA (dichloroacetate) has previously been reported to decrease cell proliferation and migration in mouse models [82]. A series of triglycerides were also identified to be decreased in the adipose secretome of the FLOT regimen receiving patients compared with treatment-naïve patients. Only TG(20:2_34:4) was found to be decreased in the adipose secretome of patients receiving CROSS compared with treatment-naïve and FLOT-receiving patients. In particular, the metabolite TG(18:0_36:3) was decreased in the adipose secretome of patients receiving the FLOT regimen compared with both treatment-naïve patients and patients receiving the CROSS regimen, whilst metabolite TG(20:2_34:4) was decreased in the adipose secretome of patients receiving the CROSS regimen compared with both treatment-naïve patients and patients receiving the FLOT regimen. Previous research has identified an increase in circulating triglycerides during neo-adjuvant chemotherapy which gradually decreases to normal levels [83]. Decreased secretion of triglycerides in the adipose secretome of patients only receiving chemotherapy raises the question of whether this decreased expression correlates with increased circulating levels and whether this can be utilised to enhance current therapies. Diminished levels of ceramides, glycosylceramides, and sphingomyelins were also observed in the adipose secretome of treated patients compared with treatment-naïve patients. Ceramide metabolism has previously been reported to induce cancer cell death through induction of cellular stress [84], and the diminished presence of ceramide and associated molecules following cancer treatment exposure could prove interesting as a therapeutic target.

Metabolite Cer(d18:1/23:0) was observed to be decreased in the adipose secretome of patients with a TRG 3 and TRG 4–5 compared with patients who had a TRG 1–2. Diminished levels of ceramides could prevent ceramide metabolism, which induces cancer cell death through the induction of cellular stress [84]. The diminished presence of ceramide in the adipose secretome of patients with more aggressive cancers could indicate that anti-cancer ceramide analogues may aid poor responding tumours [85]. Decreased expression was observed in TG(18:0_38:6) in the secretome of adipose explants derived from patients with a TRG of 1–2 and a TRG of 3 compared with patients who had a TRG of 4–5. Higher levels of TG(18:0_38:6) were observed in the adipose secretome of patients with a TRG 4–5 compared with a TRG 1–2, in addition to increased expression of TG(17:1_36:3), TG(18:0_38:6), TG(20:3_34:3), and TG(20:4_36:5) in TRG 4–5 patients compared with TRG 1–2. Higher expression of triglycerides has been linked with more aggressive cancers and decreased disease-free survival and overall survival [86]. Metabolites including PC aa C36:1 and PC aa C40:3 had increased expression in the adipose secretome of patients with TRGs of 1–2 and 4–5 compared with patients with a TRG 3. Phosphatidylcholine metabolism is linked to both cellular proliferation and cell death [87]. The decreased expression found in this study in the adipose secretome of patients with a TRG of 3 compared with patients with more regressive TRG 1–2 and more aggressive cancers with TRG scoring of 4–5 pose an interesting question of whether the adipose secretome and phosphatidylcholine metabolism may attenuate or potentiate cancer cells response to current treatment modalities.

Additionally, elevated Clavien–Dindo grade, a classification of post-operative complications was positively correlated with a series of adipose-secreted cytokines associated with pro-inflammatory response and angiogenesis including IL-6, IL-16, FLt-1, PlGF, VEGF-A, and VEGF-D. It has been previously reported that increased expression of circulating IL-6 following major abdominal surgery was associated with an increased risk of complications [88]. Furthermore, previously elevated circulating levels of VEGF have been reported following major abdominal surgery [89] and are associated with poor cancer-specific survival [90]. The elevation of these cytokines in the adipose secretome could potentiate post-op complications in these patients and may act as a future therapeutic target to ameliorate the locally based and circulating effects of these cytokines. Within this study, positive correlations were observed between increasing secretion of IL-3, IL-5, and lactic acid in the adipose secretome and patients with increasing nodal invasion. Previous research has indicated that tumour-associated leukocytes from patients with metastatic lymph nodes secreted higher levels of IL-3 and IL-5 [91]. Additionally, elevated levels of circulating lactate were previously identified in OAC patients whose tumours had metastasised to the lymph nodes compared with patients with no lymph node metastasis [92]. The potential of the adipose secretome to augment these analytes in circulation and whether this plays a pivotal role in aiding tumour metastasis to the lymph node requires further investigation to determine if these associations could be utilised for therapeutic benefit. Furthermore, within this study, decreased levels of PlGF and lactic acid in the adipose secretome were correlated with patients who showed no evidence of disease following treatment and resection. As previously mentioned, elevated lactate levels have been associated with nodal metastasis [92], and it has also been reported to be generated by cancer cells and holds critical roles in cancer cell proliferation, promoting angiogenesis and acting as a key immunosuppressive analyte [93]. This decreased expression of lactate in the adipose secretome in patients whose tumours have regressed is of interest as it raises questions on how adipose tissue plays a role in sequestering or releasing lactate and whether this could potentiate the tumour microenvironment to aid nodal invasion or recede to support cancer regression. Additionally, decreasing levels of PlGF were observed in the adipose secretome of patients whose cancers had regressed. PlGF has been reported to promote tumour desmoplasia in pancreatic mouse models with PlGF blockade., enhancing the efficacy of chemotherapy [94]. It is unknown if PlGF reduction could enhance tumour regression.

## 5. Conclusions

Overall, in this study, increases in OCR-linked oxidative phosphorylation, pro-inflammatory cytokines, and metabolites associated with aiding tumorigenesis have been identified in the most viscerally obese OAC patients and in patients with metabolic dysfunction. This now raises the question of whether targeting these altered signalling mechanisms could aid current treatment strategies. With obesity levels steadily increasing, understanding of the complex nature of adipose tissue and whether its effects can be harnessed for therapeutic gain is critically important.

## Figures and Tables

**Table 1:** Clinical demographics of patient cohort.

Patient Clinical Parameters	
**Diagnosis**	OAC = 13 OGJ = 19
**Sex**	Male = 21 Female = 11
**Obesity Status via Visceral Fat Area**	Obese = 16 Non-obese = 16
**Age at diagnosis**	51–83 (Mean = 67.83)
**Post-treatment BMI**	22.34–43 (Mean = 32.391) **Non-obese** Mean = 28.4, **Obese** Mean = 30.025 **Male** Mean = 40.282, **Female** Mean = 29.822
**Weight**	57.2–176 kg (Mean = 86.697) **Non-obese** Mean = 82.865, **Obese** Mean = 94.093 **Male** Mean = 94.72, **Female** Mean = 76.79
**Mean VFA**	22.9–485.2 (Mean = 139.404) **Non-obese** Mean = 115.192, **Obese** Mean = 167.1 **Male** Mean = 148.436, **Female** Mean = 122.872
**Metabolic Dysfunction**	*n* = 15
**High cholesterol or intervention for high cholesterol**	*n* = 21
**High blood pressure or intervention for high blood pressure**	*n* = 20
**High Triglycerides or intervention for high Triglycerides**	*n* = 4
**Diabetes**	*n* = 10
**Barrett’s oesophagus**	*n* = 17
**ASA grade (risk-stratifying system to help predict preoperative risks)**	Grade 1 → *n* = 3
	Grade 2 → *n* = 15
	Grade 3 → *n* = 10
**Clavien–Dindo classification (grading for adverse events which occur as a result of surgical procedures)**	Classification 0 → *n* = 5 Classification 1 → *n* = 4 Classification 2 → *n* = 7 Classification 3 → *n* = 7 Classification 4 → *n* = 5
**Treatment**	Naïve → *n* = 10 FLOT → *n* = 14 CROSS → *n* = 8
**Tumour Regression Grading (TRG)**	TRG 1 → *n* = 3 (CROSS *n* = 2, FLOT *n* = 1) TRG 2 → *n* = 3 (CROSS *n* = 1, FLOT *n* = 2) TRG 3 → *n* = 8 (CROSS *n* = 3, FLOT *n* = 5) TRG 4 → *n* = 3 (CROSS *n* = 2, FLOT *n* = 1) TRG 5 → *n* = 3 (CROSS *n* = 0, FLOT *n* = 3)
**Clinical Stage (T)**	T1 → *n* = 10 T2 → *n* = 3 T3 → *n* = 19
**Clinical Stage (N)**	N0 → *n* = 17 N1 → *n* = 8 N2 → *n* = 7
**Path stage (T)**	T0 → *n* = 3 T1 → *n* = 9 T2 → *n* = 4 T3 → *n* = 13 T4 → *n* = 3
**Path Stage (N)**	N0 → *n* = 17 N1 → *n* = 6 N2 → *n* = 6 N3 → *n* = 3
**Perineural Invasion**	*n* = 9
**Lymph Involvement**	*n* = 17
**Vascular Involvement**	*n* = 11
**Evidence of Disease**	*n* = 10

**Table 2:** Significant correlations associated with visceral obesity and metabolic profiles, pro-inflammatory mediators, metabolites, and lipid analysis as illustrated in Figure 1E.

	Viscerally Obese	
Factors	R	P
**OCR**	0.575376009	0.000570727
**ECAR**	0.399378641	0.023543127
**IL-17D**	0.410806922	0.041356767
**MDC**	0.381263049	0.037637047
**Asp**	−0.402873982	0.022243626
**Gln**	0.37914075	0.032351539
**Glu**	−0.392645496	0.026222596
**Cer(d18:1/23:0)**	0.390706524	0.027038963
**PC aa C42:6**	−0.481757449	0.00524064
**H1**	−0.358763864	0.043756148
**TG(14:0_36:2)**	0.370140718	0.037047715
**TG(16:0_35:3)**	0.350214483	0.049411241
**TG(16:0_38:7)**	0.466737152	0.007083324
**TG(18:2_35:2)**	0.388990152	0.027778863
**TG(18:2_38:4)**	0.357422007	0.044607706
**TG(22:5_34:3)**	0.351685392	0.048398926

## Data Availability

The data presented in this study are available on request from the corresponding author.

## Supplementary Materials

The following supporting information can be downloaded at: https://www.mdpi.com/article/10.3390/cancers15061681/s1, Table S1: Significant correlations associated with Figure 2E between experimental data and factors associated with metabolic dysfunction and systemic disease; Table S2: Significant correlations associated with Figure 3E for clinical correlations with neo-adjuvant treatment, chemotherapy only, chemoradiotherapy and experimental data; Table S3: Significant correlations associated with Figure 3E for clinical correlations with differentiation, lymph involvement, venous involvement and perineural involvement; Table S4: Significant correlations associated with Figure 4D for clinical correlations with tumour regression grade, clinical tumour stage and clinical nodal stage; Table S5: Significant correlations associated with Figure 4D for clinical correlations with pathological tumour stage, pathological nodal stage and no evidence of disease
