# Supplementary material for: Energy Metabolism, Metabolite, and Inflammatory Profiles in Human Ex Vivo Adipose Tissue Are Influenced by Obesity Status, Metabolic Dysfunction, and Treatment Regimes in Patients with Oesophageal Adenocarcinoma
Source: Cancers (Basel). 2023 Mar 9;15(6):1681. doi: 10.3390/cancers15061681 (PMC10046380; doi:10.3390/cancers15061681)
Supplement: Supplementary file 1 [file cancers-15-01681-s001.zip › cancers-2243206-supplementary.pdf]

Supplemental Table S1. Significant correlations associated with Figure 2. E. between experimental data and factors associated with metabolic dysfunction and systemic disease.

	Metabolic Dysfunction	Barrett's Oesophagus	Smoking History	ASA grade	Clavien-Dindo Grade
**Factors**			**R number (p value)**		
**Flt-1**					0.448 (0.019)
**GM-CSF**		-0.44 (0.028)			
**IFN-γ**		-0.5 (0.025)			
**IL-16**					0.436 (0.023)
**IL-1α**		-0.506 (0.016)			
**IL-22**	0.436 (0.026)				
**IL-6**					0.394 (0.042)
**MIP-1α**		-0.485 (0.022)			
**PIGF**					0.501 (0.008)
**Tie-2**				0.525 (0.037)	
**TSLP**		-0.404 (0.045)			
**VEGF-A**					0.382 (0.049)
**VEGF-D**					0.475 (0.022)
**Gly**	0.383 (0.03)				
**His**	0.414 (0.018)				
**Phe**	0.4 (0.023)				
**Trp**	0.363 (0.041)				
**HCys**	0.424 (0.016)				
**GUDCA**	0.418 (0.017)				
**beta-Ala**	0.421 (0.017)				
**Cer(d18:1/23:0)**		-0.422 (0.032)	-0.376 (0.037)		
**DG(18:3_18:3)**		-0.39 (0.049)			
**Cer(d18:0/24:0)**	0.379 (0.032)				
**PC aa C38:3**					0.4 (0.035)
**PC aa C40:3**		-0.434 (0.027)			
**PC ae C34:1**	0.469 (0.007)				
**Hex3Cer(d18:1/24:1)**				-0.409 (0.038)	
**HexCer(d18:1/ 18:0)**	0.392 (0.026)				
**TG(16:0_38:6)**	0.434 (0.013)		0.433 (0.015)		
**TG(16:0_40:6)**				-0.418 (0.034)	
**TG(16:1_32:0)**		-0.4 (0.043)			
**TG(16:1_36:4)**				-0.434 (0.027)	
**TG(17:1_36:3)**	0.429 (0.014)				
**TG(18:0_38:6)**	0.382 (0.031)				
**TG(18:2_32:2)**					-0.46 (0.014)
**TG(18:2_38:4)**	0.46 (0.008)				
**TG(18:3_35:2)**	0.481 (0.005)				
**TG(20:3_34:0)**		-0.418 (0.034)			
**TG(20:4_33:2)**				-0.45 (0.021)	
**TG(20:4_36:3)**			-0.545 (0.002)		
**TG(22:6_34:3)**			-0.419 (0.019)		-0.377 (0.048)

Supplemental Table S2. Significant correlations associated with Figure 3. E. for clinical correlations with neo-adjuvant treatment, chemotherapy only, chemoradiotherapy and experimental data.

	Neo-adjuvant treatment	Chemotherapy only	Chemoradiotherapy
**Factors**		**R number (p value)**	
**IL-12p40**		0.536 (0.006)	
**IL-22**		0.433 (0.034)	
**IL-31**		0.659 (0.004)	
**IL-7**		0.383 (0.049)	
**sICAM-1**		0.381 (0.041)	
**sVCAM-1**		0.39 (0.037)	
**DCA**		-0.387 (0.034)	0.455 (0.012)
**GUDCA**		0.366 (0.047)	-0.364 (0.048)

**Cer(d16:1/22:0)**	-0.391	(0.033)				
**Cer(d18:1/23:0)**			-0.376	(0.04)		
**DG(18:1_20:1)**	0.393	(0.032)				
**PC aa C34:3**			-0.387	(0.035)		
**PC aa C42:1**	0.437	(0.016)	0.453	(0.012)		
**SM (OH) C16:1**			-0.412	(0.024)	0.446	(0.013)
**SM (OH) C22:1**	-0.386	(0.035)	-0.432	(0.017)		
**TG(14:0_36:2)**			-0.499	(0.005)	0.48	(0.007)
**TG(16:0_30:2)**			-0.483	(0.007)		
**TG(16:0_34:2)**			-0.397	(0.03)		
**TG(16:0_34:3)**			-0.472	(0.008)		
**TG(16:0_36:3)**			-0.403	(0.027)		
**TG(16:1_32:0)**			-0.395	(0.031)		
**TG(16:1_34:1)**	-0.423	(0.02)	-0.459	(0.011)		
**TG(16:1_34:2)**	-0.375	(0.041)	-0.459	(0.011)		
**TG(16:1_36:4)**			-0.388	(0.034)		
**TG(16:1_36:5)**					0.465	(0.01)
**TG(18:0_36:3)**			-0.491	(0.006)		
**TG(18:1_32:1)**			-0.494	(0.006)		
**TG(18:1_32:3)**					0.432	(0.017)
**TG(18:2_32:1)**			-0.432	(0.017)		
**TG(18:3_32:1)**			-0.606	(0.0003)	0.527	(0.003)
**TG(20:2_34:4)**					-0.484	(0.007)
**TG(20:4_36:3)**					0.423	(0.02)
**TG(22:5_34:3)**					0.395	(0.031)

Supplemental Table S3. Significant correlations associated with Figure 3. E. for clinical correlations with differentiation, lymph involvement, venous involvement and perineural involvement.

	**Differentiation**		**Lymph Involvement**		**Venous Involvement**		**Perineural Involvement**	
**Factors**	**R number (p value)**							
**OCR**			0.641	(0.001)				
**IL-3**			0.677	(0.004)				
**IL-5**			0.598	(0.011)				
**PIGF**							-0.558	(0.013)
**SAA**							-0.462	(0.047)
**Tie-2**							-0.806	(0.0001)
**VEGF-D**							-0.553	(0.021)
**ADMA**					0.422	(0.018)		
**Orn**					0.366	(0.043)		
**Putrescine**	-0.371	(0.047)						
**Lac**			0.521	(0.013)				
**Cer(d16:1/22:0)**			-0.728	(0.0001)				
**Cer(d16:1/24:0)**					0.422	(0.018)		
**Cer(d18:2/24:0)**							-0.551	(0.014)
**DG(16:1_18:2)**			-0.44	(0.04)				
**lysoPC a C18:0**							-0.521	(0.022)
**PC aa C34:3**	-0.391	(0.036)						
**Hex2Cer(d18:1/14:0)**			0.501	(0.018)				
**HexCer(d18:1/18:1)**							-0.562	(0.012)
**Hypoxanthine**					0.392	(0.029)		
**TG(16:0_33:2)**	-0.473	(0.01)						
**TG(16:0_38:3)**			-0.592	(0.004)				
**TG(16:0_38:7)**	-0.374	(0.046)						
**TG(16:0_40:6)**			-0.588	(0.004)				
**TG(16:0_40:7)**							-0.66	(0.002)
**TG(16:1_34:2)**	-0.38	(0.042)						
**TG(16:1_36:1)**	-0.425	(0.022)						
**TG(16:1_36:2)**			-0.436	(0.043)				

**TG(16:1_36:5)**					0.47	(0.042)
**TG(17:1_36:3)**			-0.431	(0.045)		
**TG(18:1_33:3)**					0.489	(0.034)
**TG(18:1_35:3)**			-0.47	(0.027)		
**TG(18:3_34:1)**			-0.535	(0.01)		
**TG(20:4_34:2)**			-0.452	(0.035)		
**TG(20:4_36:5)**	-0.55	(0.002)	0.49	(0.02)		
**TG(22:6_32:0)**	-0.592	(0.001)				

Supplemental Table S4. Significant correlations associated with Figure 4. D for clinical correlations with tumour regression grade, clinical tumour stage and clinical nodal stage.

	Tumour Regression Grade		Clinical Tumour Stage		Clinical Nodal Stage	
**Factors**	R number (p value)					
**IL-12p40**	0.544	(0.02)				
**IL-17D**					-0.548	(0.004)
**IL-1α**	0.435	(0.049)				
**IL-22**					0.521	(0.006)
**IL-5**	0.639	(0.006)			0.434	(0.043)
**Ala**	0.417	(0.048)				
**Lys**	0.436	(0.037)				
**Tyr**	0.452	(0.03)				
**Cer(d16:1/23:0)**	-0.439	(0.036)				
**Cer(d18:1/23:0)**	0.516	(0.012)				
**DG(18:0_20:4)**	0.455	(0.029)			0.403	(0.022)
**DG(18:3_18:3)**			0.411	(0.02)	0.37	(0.037)
**PC aa C40:3**			0.424	(0.016)		
**Hex2Cer(d18:1/24:0)**	-0.491	(0.017)				
**TG(14:0_38:5)**			-0.353	(0.047)		
**TG(16:0_35:3)**	0.496	(0.016)			0.371	(0.037)
**TG(18:2_35:3)**	0.617	(0.002)				
**TG(18:3_34:0)**					0.37	(0.037)
**TG(20:0_32:3)**					0.396	(0.025)
**TG(20:4_36:3)**	0.511	(0.013)				
**TG(22:6_34:3)**			0.351	(0.049)		

Supplemental Table S5. Significant correlations associated with Figure 4. D for clinical correlations with pathological tumour stage, pathological nodal stage and no evidence of disease.

	Pathological Tumour Stage		Pathological Nodal Stage		No evidence of disease	
**Factors**	R number (p value)					
**Eotaxin**			0.37	(0.04)		
**GM-CSF**			0.491	(0.005)		
**IL-15**			0.476	(0.007)		
**IL-1RA**			0.578	(0.001)		
**IL-1α**			0.64	(0.0003)		
**IL-1β**			0.364	(0.044)		
**IL-22**			0.482	(0.013)		
**IL-23**	0.695	(0.006)				
**IL-3**			0.425	(0.049)		
**IL-5**	0.449	(0.036)	0.605	(0.003)		
**IP-10**			0.486	(0.006)		
**MCP-1**			0.384	(0.033)		
**MIP-1α**			0.423	(0.025)		
**MIP-1β**			0.42	(0.019)		
**PIGF**					-0.447	(0.012)
**sICAM-1**			0.381	(0.035)		
**sVCAM-1**			0.4	(0.026)		
**TNF-α**			0.382	(0.034)		
**Kynurenine**			0.376	(0.034)		

**Met-SO**			0.46	(0.008)		
**Lac**	0.358	(0.045)				
**Cer(d16:1/22:0)**					-0.409	(0.02)
**Cer(d18:1/16:0)**					-0.371	(0.037)
**Cer(d18:1/23:0)**	0.372	(0.036)				
**Cer(d18:1/24:1)**					-0.436	(0.013)
**DG(16:1_18:2)**	-0.372	(0.036)				
**DG(18:0_20:4)**			0.384	(0.03)		
**lysoPC a C18:0**	0.365	(0.04)				
**PC aa C32:0**					-0.402	(0.023)
**PC aa C34:1**					-0.467	(0.007)
**PC aa C36:4**					-0.398	(0.024)
**PC ae C32:1**					-0.405	(0.022)
**PC ae C34:2**			-0.351	(0.049)		
**Hex2Cer(d18:1/14:0)**			0.362	(0.042)		
**SM C24:1**					-0.385	(0.029)
**TG(14:0_38:5)**					-0.438	(0.012)
**TG(16:0_38:6)**	-0.378	(0.033)				
**TG(16:0_38:7)**					-0.359	(0.044)
**TG(16:0_40:6)**					-0.388	(0.028)
**TG(18:1_38:5)**					-0.354	(0.047)
**TG(18:1_38:6)**			-0.368	(0.038)		
